# A Public Mental Health Study Among Iraqi Refugees in Sweden: Social Determinants, Resilience, Gender, and Cultural Context

**DOI:** 10.3389/fsoc.2021.551105

**Published:** 2021-04-26

**Authors:** Önver A. Çetrez, Valerie DeMarinis, Maria Sundvall, Manuel Fernandez-Gonzalez, Liubov Borisova, David Titelman

**Affiliations:** ^1^Faculty of Theology, Uppsala University, Uppsala, Sweden; ^2^Public Mental Health Promotion Research Area, Innlandet Hospital Trust, Brumunddal, Norway; ^3^Department of Public Health and Clinical Medicine, Umeå University, Umeå, Sweden; ^4^Department of Learning, Informatics, Management and Ethics, National Centre for Suicide Research and Prevention of Mental Ill-Health, Karolinska Institutet, Stockholm, Sweden; ^5^Unit for Transcultural Psychiatry, Department of Psychiatry, Uppsala University Hospital, Uppsala, Sweden; ^6^Department of Sociology, Uppsala University, Uppsala, Sweden

**Keywords:** Iraqi, refugees, acculturation, mental health, trauma, social support, perceptions of illness, resilience

## Abstract

This public mental health study highlights the interactions among social determinants and resilience on mental health, PTSD and acculturation among Iraqi refugees in Sweden 2012-2013.

**Objectives:** The study aims to understand participants' health, resilience and acculturation, paying specific attention to gender differences.

**Design:** The study, using a convenience sampling survey design (*N* = 4010, 53.2% men), included measures on social determinants, general health, coping, CD-RISC, selected questions from the EMIC, PC-PTSD, and acculturation.

**Results:** Gender differences and reported differences between life experiences in Iraq and Sweden were strong. In Sweden, religious activity was more widespread among women, whereas activity reflecting religion and spirituality as a coping mechanism decreased significantly among men. A sense of belonging both to a Swedish and an Iraqi ethnic identity was frequent. Positive self-evaluation in personal and social areas and goals in life was strong. The strongest perceived source of social support was from parents and siblings, while support from authorities generally was perceived as low. Self-rated health was high and the incidence of PTSD was low. A clear majority identified multiple social determinants contributing to mental health problems. Social or situational and emotional or developmental explanations were the most common. In general, resilience (as measured with CD-RISC) was low, with women's scores lower than that of men.

**Conclusions:** Vulnerability manifested itself in unemployment after a long period in Sweden, weak social networks outside the family, unsupportive authorities, gender differences in acculturation, and women showing more mental health problems. Though low socially determined personal scores of resilience were found, we also identified a strong level of resilience, when using a culture-sensitive approach and appraising resilience as expressed in coping, meaning, and goals in life. Clinicians need to be aware of the risks of poorer mental health among refugees in general and women in particular, although mental health problems should not be presumed in the individual patient. Instead clinicians need to find ways of exploring the cultural and social worlds and needs of refugee patients. Authorities need to address the described post-migration problems and unmet needs of social support, together comprising the well-established area of the social determinants of health.

## Introduction

In the Roadmap for Mental Health in Europe (ROAMER) article, on behalf of the ROAMER consortium funded by the European Commission, under the Seventh Framework Programme (Forsman et al., 2015), the following three priorities are particularly relevant (Forsman et al., 2015, p. 251–252):

1. Positive mental health and well-being and protective factors should be increasingly addressed in public mental health research.
2. Public mental health research should build on interdisciplinary perspectives in order to understand the complexity of mental health.
3. Studies should strengthen the understanding of the cultural factors (i.e., ethnicity, religion and value systems, and nationality) relevant for public mental health.

Following these recommendations, we in this study explore aspects of the migration process of Iraqi refugees who arrived in Sweden between 2010 and 2013. In accord with the ROAMER priorities, our aim was to elucidate enabling factors (social and cultural, and other resilience factors) as well as conceivable barriers to mental health and acculturation in this cohort. The study's theoretical foundation therefore rests on a two-domain model of mental health where mental ill-health and subjective well-being are distinct constructs (Patalay and Fitzsimons, 2016).

**Figure 1:**
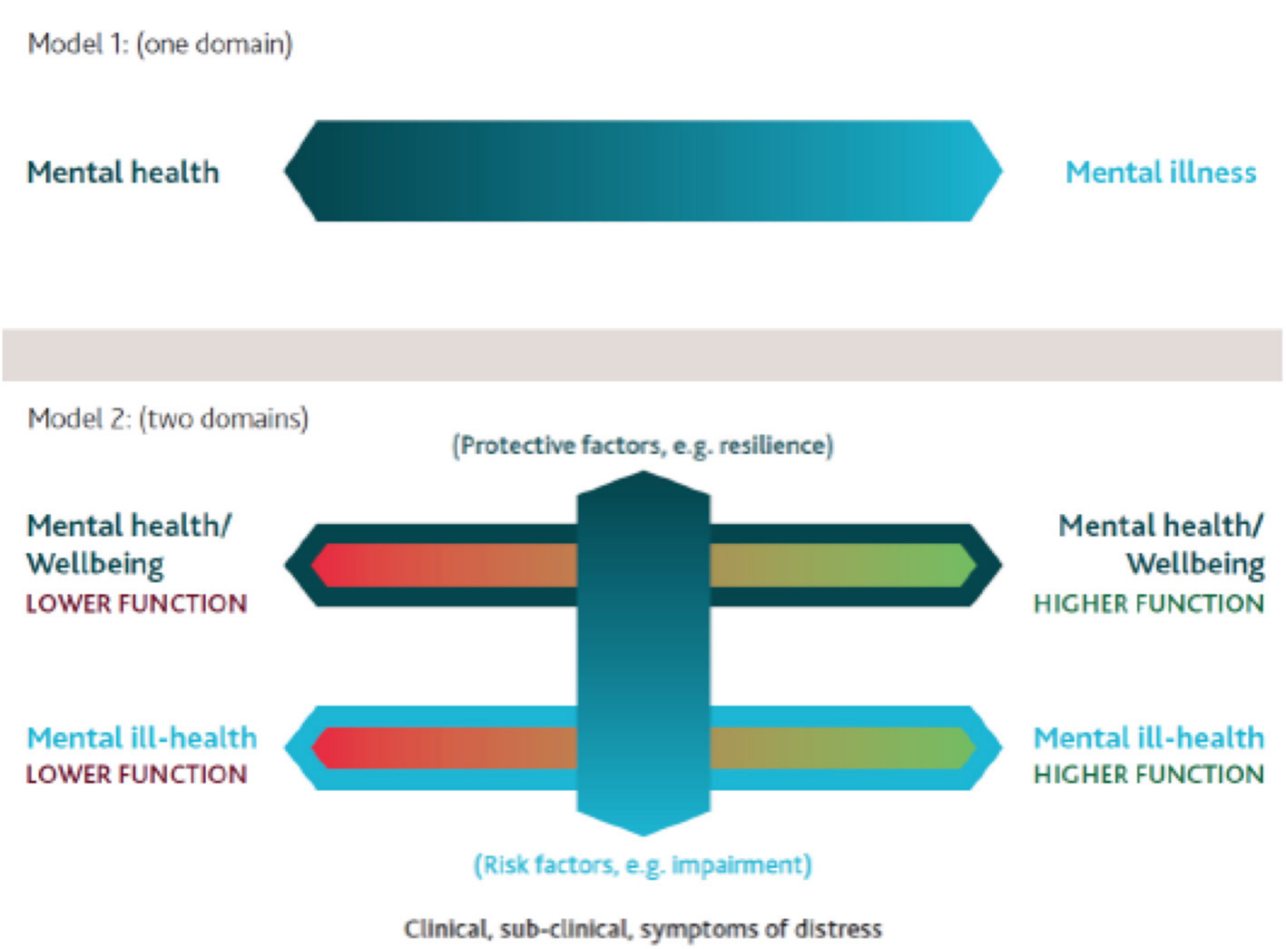
Two-domain-model of mental health. Modified from (Boyd-MacMillan and DeMarinis, 2020).

The two-domain model as depicted in Figure 1 permits a more complete understanding of the different domains of mental health (domain 1—mental health/mental illness, and domain 2—mental health/well-being) and focuses on the numerous interacting factors between them that can affect actual daily function. The model is fluid, and reflects the growing evidence of interaction between the two domains (Kalra et al., 2012; Patalay and Fitzsimons, 2016; DeMarinis and Boyd-MacMillan, 2019; Boyd-MacMillan and DeMarinis, 2020). Addressing the important critique that Betancourt and Khan (2008) raise that the focus on trauma alone has resulted in inadequate attention to factors associated with resilient mental health outcomes, our application of the two-domain model acknowledges the negative mental health consequences of war-related and other forms of violence and loss as well as other determinants, and allows for reflecting on multiple theoretical areas needed to investigate mental health.

**Figure 2:**
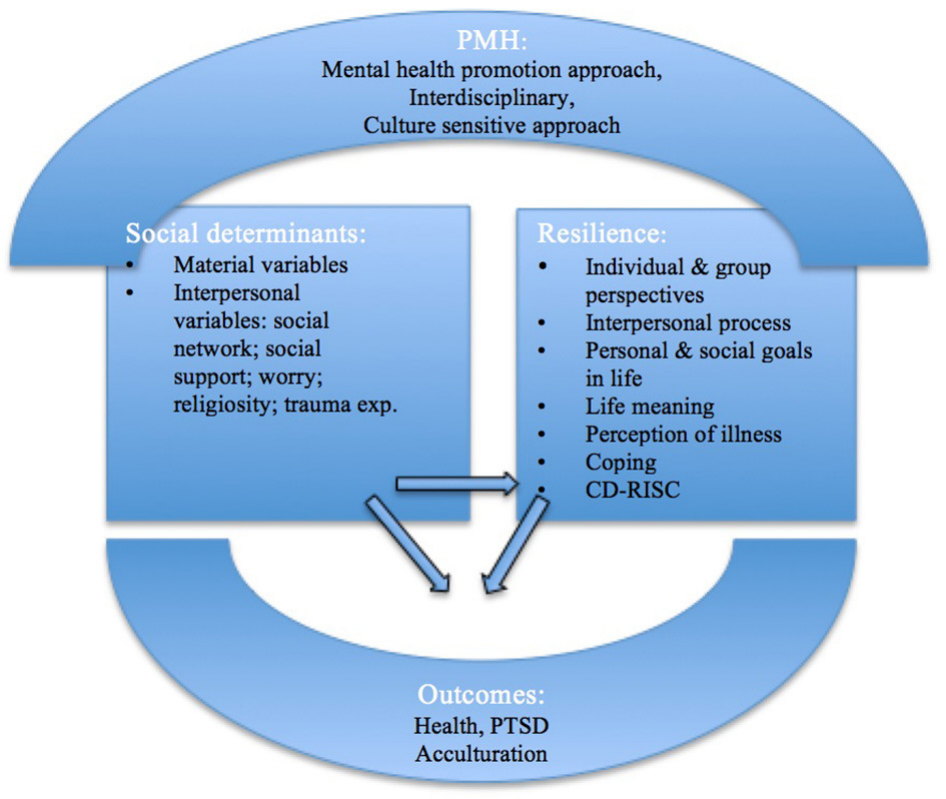
Theoretical overview with a public mental health approach to social determinants, resilience and outcomes. Original figure developed by authors. PMH, Public Mental Health; CD-RISC, Connor-Davidson Resilience Scale; PTSD, Post-traumatic stress disorder.

In Figure 2 we describe the theoretical framework used and the relationships between the theoretical perspectives included in this framework.

### Social Determinants of Health

Adverse social conditions, including poverty, unemployment, housing problems, gender-based discrimination, social stratification and unfairness, and lack of social support may lead to health inequity and hinder people from having “the freedom to lead flourishing lives” (Marmot, 2007). Thinking about the social realities of refugees, it is relevant to take into account stages of their resettlement: premigration, during migration, and post-migration. As seen in Figure 2, social determinants can be characterized as either material variables (a physically safe environment, housing, healthcare, education, economy, and policies) or interpersonal ones (e.g., a sense of cultural, ethnic, or religious belonging, social network and support, trauma experiences, discrimination, and social status) (Hynie, 2018; Cetrez et al., 2020b).

#### Material Variables

For decades, segments of the Iraqi population suffered from financial restraints, unemployment, internal displacement, conscriptions for war, lack of food and medicine, and destruction of healthcare infrastructure (Jamil et al., 2010; Warda et al., 2018).

Between 2000 and 2016 Sweden received a total of 90,217 Iraqi asylum seekers, 20,858 of whom arrived in 2015 (Migration Agency [Migrationsverket], 2017). A report on Swedish integration policies, practices, and experiences (Cetrez et al., 2020a) emphasizes recent drastic changes of legislative measures, including a shift of focus from integration to individual establishment. While newcomers are welcome to participate in different educational programs, segregation in housing has become a major challenge. In general, access to health care services is relatively unproblematic.

#### Interpersonal Variables

Earlier studies have found that the weakening of social networks and lack of social support affect the mental health and quality of life of refugees (Gorst-Unsworth and Goldenberg, 1998; Laban et al., 2005; Gerritsen et al., 2006; Sundvall et al., 2020). In a review of studies on resettled war refugees, the impact of low social support was especially noticeable in association with depression (Bogic et al., 2015). Tinghög et al. (2016) demonstrated a strong association between low social support and symptoms of anxiety and depression as well as low levels of well-being among refugees from Eritrea, Somalia, and Syria living in Sweden.

Refugees often report high levels of traumatic experiences in the premigration period. In a study on Iraqi male migrants to the UK 1990-93, Gorst-Unsworth and Goldenberg (1998) reported that 65% of these refugees had experienced torture during periods of detention. In a recent study of refugees in the USA, those from Iraq had often reported either having experienced torture, violence, or imprisonment themselves, or witnessed the torture or violence (Schlaudt et al., 2020).

### Resilience

A public mental health promotion approach focuses on protective and salutogenic factors that contribute to resilience (DeMarinis, 2014). These social determinants operate in both pre- and post-migration contexts (Mawani, 2014). Resilience denotes the personal qualities that enable an individual to thrive in the face of adversity (Connor and Davidson, 2003); it is associated with improvements in both physical and mental health (Connor et al., 1999). Resilience may also be viewed as a measure of successful coping with stress (Connor and Davidson, 2003). *Coping* can be defined as a process through which individuals attempt to understand and deal with important demands in their lives (Ganzevoort, 1998), or as a search for meaning in difficult times (Pargament, 1997). *Meaning-making* is a general human characteristic, and *existential meaning* refers to an individual's or a group's most essential meaning-making activity (DeMarinis, 2014). A concept related to resilience, ‘hardiness’ has been used as an index of mental health (Ramaniah et al., 1999). Both hardiness and resilience have been shown to protect against developing chronic post-traumatic stress disorder (PTSD) after traumatic experiences (Waysman et al., 2001).

A person's resilience is, however, not only an individual process but also an interpersonal one, that is, a human resource that develops and thrives in a culturally defined group- and community context (Kirmayer et al., 2011). Ungar's (2008) definition of resilience includes the processes of social navigation and negotiation:

In the context of exposure to significant adversity, whether psychological, environmental, or both, resilience is both the capacity of individuals to navigate their way to health-sustaining resources, including opportunities to experience feelings of well-being, and a condition of the individual's family, community and culture to provide these health resources and experiences in culturally meaningful ways (p. 225).

Further, Mawani (2014) showed an association between religious/spiritual support and positive health outcomes, indicating that refugees prefer finding support within their own group for reasons of language, connection, and trust. According to Walsh (2006), both family and community provide supportive belief systems, organizational patterns, and communication processes; your own culture is at the same time a source of social support and of social pressure. Additionally, Panter-Brick and Eggerman (2012) underscored that an ecological approach to resilience indicates “a sense of meaning-making that orders the world and gives coherence to the past, present, and the future” (p. 385).

Kirmayer et al. (2011) objected to resilience research merely focusing on the inverse of risk factors and favored an approach to resilience as a dynamic process of adaptation and transformation. Taking this point to heart, we include a focus on the refugee's *perception of illness* and help-seeking behavior.

Following a society's value system adds to a person's social resilience in that societal context. In contemporary Sweden, gender equality, secular-rational worldviews, and a high esteem of self-expression are predominant values (Welzel, 2013; DeMarinis, 2014). If a minority group or individuals in such groups espouse and practice a value system at odds with the larger societal system, then drops in both individual and social resilience levels can occur.

### Outcomes

#### Health

A study of self-perceived health among newly arrived Iraqis to Sweden 2007–2008 showed that 55% rated their general health as good or very good, with no significant difference between genders, age groups, or individuals with different levels of education (Sundell Lecerof, 2010). However, 16% of the studied migrants reported poor or very poor general health and 37% reported reduced mental well-being, both rates twice as high as that in the general Swedish population; 29% responded that they often felt stressed in everyday life. In a study on Iraqi male refugees in the UK by Gorst-Unsworth and Goldenberg (1998), 10.7% of the participants evinced PTSD. Taylor et al. (2014) reported chronic health conditions, emotional stress, and depression among half of an Iraqi refugee sample residing in the U.S. (*N* = 366) 2007–2010. In a mental health screening of 8,149 refugees of different nationalities in Kentucky, 22.13% screened positively for emotional distress on Refugee Health Screener-15 (RHS-15), predicting depression, anxiety, and PTSD. Almost half of the Iraqi participants had positive scores; higher age and females predicted positive scores. A systematic literature review by Bogic et al. (2015) showed that higher exposure to traumatic experiences and post-migration stress were the most common factors consistently associated with higher rates of mental disorders in war-refugees.

In a meta-analysis of 20 studies, Fazel et al. (2005) found that about 10% of adult refugees in Western countries suffer from PTSD. The prevalence of PTSD specifically among Iraqi refugees in different countries, however, varies strongly, between 14% (Kira et al., 2009), 28% (Jamil et al., 2005), 31% (Taylor et al., 2014), 37% (Söndergaard et al., 2004), 43% (Craig et al., 2006), and 83% (Daud et al., 2008). Kira et al. (2009) found that PTSD was more prevalent in women than in men and in individuals who were married, divorced, widowed, or adolescents than in those who were single or temporarily separated. The same study showed a high prevalence of PTSD among the elderly (70+) and people aged 41–50 as well as an association between PTSD and length of stay in the new country, and with low education.

Several studies demonstrate an association between post-migration experiences, for example, a long period as an asylum seeker (Laban et al., 2004) or being in detention during the asylum process (Steel et al., 2006) and high scores of PTSD and other mental disorders. In a study on Iraqi-Mandaean refugees in Australia by Nickerson et al. (2009), a reversed sequence of determination was noted: PTSD predicted a subsequent fear of cultural extinction.

#### Acculturation

Acculturation refers to the acquisition of a second culture and the process of perceiving new practices and behaviors in the encounter between two cultural groups (Rudmin, 2009), with outcomes such as integration, separation, assimilation and marginalization (Berry, 2006). Acculturation includes coping with social and psychological conflicts (intrapsychic or interpersonal), e.g., when cultural norms clash, or when gender roles and relationships are renegotiated and women take novel positions of power in their family (Suarez-Orozco, 2000; Nelson et al., 2016). Rudmin (2009) documented two critical social determinants of acculturation: the migrant's socioeconomic situation and his or her perception of discrimination in society.

An important field of acculturation research relates education and language skills to measures of health, e.g., experienced stress and depression (Wrobel et al., 2009; Hahn and Truman, 2015). Van Tubergen (2010) elucidated the links between post-migration language acquisition and pre-migration factors such as schooling, age at the time of migration, geographic mobility, time spent in refugee reception facilities, completion of integration courses and other education, intention to remain in the host country, and complex health problems. Other research has demonstrated that negative health outcomes are linked to refugee- or temporary resident status (Steel et al., 2006), pre-migration traumatic events and continuous high levels of stress (Rian and Hodge, 2010), psychopathology, and post-migration living problems (Laban et al., 2005), abuse (Padela and Heisler, 2010), and experiences of discrimination, detention, dispersal, destitution, delayed decisions on asylum, denial of the right to work, or denial to healthcare—*the seven D's* (McKenzie et al., 2014).

Studies on identity and acculturation, including that of Iraqi minority subgroups (Cetrez, 2005, 2011, 2015) cautions against a simplistic understanding of belonging as an either-or identification with a given culture. Allegiance to multiple groups is promoted by what Collie et al. (2010) describe as a mindful, strategic, and contextual identity negotiation, as is the co-existence within one person of different ethnic identities (Sirina et al., 2008).

### Aim

The aim of this study was to explore the social and psychological determinants of health, especially mental health, and resilience and acculturation outcomes in a population of resettled Iraqi refugees who arrived in Sweden in the years 2000-2013. We also wished to investigate gender differences and situate our findings in the Swedish societal context.

## Method

### Sample

The studied group of refugees was a convenience sample, recruited through written information material, key persons in community networks, associations, organizations, schools, and workplaces. Our inclusion criteria were being Iraqi, having moved to Sweden between 2000 and 2013, and residing in the cities of Stockholm, Uppsala or Södertälje. Table 1 presents the sample, 410 individuals of whom 218 were men (53.2%) and 192 women (46.8%). The women (*M* = 34.27, *SD* = 14.27) were significantly younger than the men (*M* = 39.98, *SD* = 16.14) (*p* < 0.001). Equal fractions of the respondents (33–34%) resided in each of the three cities.

**Table 1:** Frequency and percentage for age, residence, and arrival, by gender.

	**Men (%)**	**Women (%)**	**Total (%)**	**Missing**
Overall sample	218 (53.2)	192 (46.8)	410	
Age group***				0
18–24	45 (20.6)	66 (34.4)	111 (27.1)	
25–34	54 (24.8)	53 (27.6)	107 (26.1)	
35–47	51 (23.4)	36 (18.8)	87 (21.2)	
48+	68 (31.2)	37 (19.3)	105 (25.6)	
Current city of residence				0
Södertälje	79 (36.2)	59 (30.7)	138 (33.7)	
Uppsala	66 (30.3)	71 (37)	137 (33.4)	
Stockholm	73 (33.5)	62 (32.3)	135 (32.9)	
Median year of arrival in Sweden	2006	2006	2006	0

*Chi-square tests for gender differences*,

*** *p < 0.01*.

### Instruments

The survey comprised items linked to the outlined theoretical themes. To explore the material social determinants, we included items on education, employment, economy, and physical safety. For the interpersonal variables, we included items on Swedish language proficiency, aspects of religion, and social support (emotional, concrete, informational, or spiritual) (Tracy and Whittaker, 1990; Balboni et al., 2007), and trauma experience. There were also items on everyday worries, both in Iraq and in Sweden. To document individual and cultural dimensions of resilience we included items linked to meaning of life, life goals, coping, and perceptions of illness (the latter from the Explanatory Model Interview Catalog, EMIC, Weiss, 1997). Resilience was also gauged with items from The Connor-Davidson Resilience Scale (CD-RISC 2) (Davidson, 2018, Vaishnavi et al., 2007). To evaluate mental-health, we included items on self-perceived health from the PC-PTSD screen (Prins et al., 2003). Acculturation was explored through items on identity (Cetrez, 2005), in addition to questions about living conditions in Iraq, compared to Sweden (see Appendix for the full questionnaire).

### Procedure

The questionnaire was pretested (focusing on concepts, scales, language, and sensitivity, and refined/shortened accordingly) with different subgroups of respondents: two female and two male representatives from each of the three main religious denominations—Christian, Muslim, and Mandaean (in total 12 persons). Surveys were conducted in Arabic, English or Swedish, depending on the preference of the participants. It took on the average 30–40 min to complete the survey, but longer, up to 90 min, for individuals who needed help in reading and writing. Although designed as a self-administered questionnaire, most of the surveys were filled out in the presence of an Arabic-speaking research assistant (who provided language support). The setting for filling in the questionnaire was at the choice of the study person, for example, at school, work, cafés, and similar.

### Data Analyses

The survey study data were analyzed with SPSS (version 27), limited to descriptive analyses using gender and country as independent variables.

### Ethical Considerations

Participants were informed of the availability, through our auspices, of a psychiatric consultant, if needed. An interpreter would also be available as needed. Ethical approval was granted by the Uppsala Regional Ethical Review Board (reg. no. Dnr 2011/394).

## Results

### Social Determinants

As shown in Table 2, the respondents were mostly married or in a partnered relationship (59.6%), with two-fifths having children who lived at home. A majority (55.4% of the total sample) had a university degree. In this subgroup two-thirds had completed their education in Iraq, a third had done so in Sweden. The majority of the total sample (75.9%) had an occupation of some sort (including unpaid work). The rate of unemployment was significantly higher among men than women (see Table 2). Of those who were unemployed, two-thirds received support from municipal social services, and a tenth received government benefits. During the last 12 months, two-fifths of the unemployed had had difficulties in making ends meet: paying bills for food, rent, etc. A majority of the respondents reported their competency in Swedish as strong. Although most of them did not speak Swedish at home, they spoke and wrote Swedish at work, on the internet, and with friends.

**Table 2:** Social determinants, by gender (*N* = 410).

	**Men**		**Women**		**Total**		**Missing**
	***N***	**%**	***N***	**%**	***N***	**%**	
Relationship status: in a relationship*	138	63.6	105	45	243	59.6	2
Children at home*	96	44.04	74	38.54	170	41.46	0
Education: University	118	54.38	108	56.54	226	55.39	2
Unemployed**	60	28.4	36	19.3	96	24.1	12
Economic difficulty**	101	46.3	69	36.1	170	41.6	1
Strong Swedish proficiency	138	63.9	129	67.9	267	65.8	4
Use Swedish at home	34	15.96	37	20	71	17.84	12
Use Swedish at work	139	88.54	128	90.14	267	89.3	111
Use Swedish in free time (friends, Internet, other free time)	157	76.96	150	81.52	307	79.12	22

*Chi-square tests for gender differences*,

** *p < 0.05*,

* *p < 0.1*.

Table 3 covers life meaning, coping, and worries, both in Iraq and Sweden. Most respondents reported that worrying about family, friends and safety was the worst and that they were relieved when these concerns subsided. This experience of positive change was significant both for men and women. Yet, worries about work and studies significantly increased over time for both men and women. Overall, significant gender differences were present in connection with worrying about family: men tended to worry more than women (in Iraq: *p* = 0.042, in Sweden: *p* = 0.065). A majority of the total sample (app. 91%) felt either “very” (69%) or “somewhat” (22%) safe at home as well in their neighborhood (86%).

**Table 3:** Descriptive statistics for life meaning, coping and worries, by gender and by country.

	**Men**							**Women**						
	**Iraq**			**Sweden**				**Iraq**			**Sweden**			
	**N**	**Mean**	**SD**	**N**	**Mean**	**SD**	**M diff**	**N**	**Mean**	**SD**	**N**	**Mean**	**SD**	**M diff**.
**How much did/does … help you to make sense/give your life meaning during your most stable period in Iraq or now in Sweden?**														
Family	206	9.10	2.00	194	9.11	1.48	−0.01	182	9.40	1.43	179	9.21	1.57	0.19***
Friends	174	7.49	2.46	170	7.27	2.47	0.22	147	7.22	2.62	161	7.66	2.48	−0.44
Religion & spirituality	168	7.31	3.06	159	7.22	3.13	0.09	151	8.02	2.56	151	8.07	2.63	−0.05
Work & school	163	7.67	2.53	161	7.54	2.59	0.13	151	8.15	2.32	157	8.10	2.51	0.05
**To what extent did/does … help you in coping with any difficult situation you are facing during your most stable period in Iraq or now in Sweden?**														
Family	191	9.47	1.26	190	9.07	1.94	0.40**	172	9.41	1.42	176	9.30	1.44	0.11
Friends	161	7.71	2.23	166	7.43	2.56	0.28	134	7.13	2.77	152	7.49	2.57	−0.37
Religion & spirituality	150	7.66	2.77	153	7.22	3.02	0.44*	137	8.01	2.67	146	8.16	2.62	−0.16
Work & school	147	7.31	2.57	152	6.97	2.66	0.34	130	7.06	2.69	146	7.45	2.68	−0.38*
Being out in nature	127	6.51	3.09	152	8.01	2.30	−1.49***	118	6.00	3.32	153	8.18	2.35	−2.18***
**What worried/worries you most, during your most difficult time in Iraq and now in Sweden**														
Family	139	8.73	2.46	155	7.61	3.12	1.12***	125	8.02	3.17	130	6.86	3.66	1.15***
Friends	124	6.43	2.72	127	5.89	3.02	0.54***	104	6.11	2.95	115	5.34	3.30	0.77*
Work & school	115	6.77	3.00	153	7.67	2.84	−0.91**	110	6.65	3.07	149	7.50	2.99	−0.85*
Safety	183	8.95	2.40	106	5.13	3.40	3.82**	161	8.89	2.30	115	4.89	3.45	4.01***

*Chi–square tests for country differences*,

*** *p < 0.01*,

** *p < 0.05*,

* *p < 0.1*.

*Scale from 1 (Not at all) to 10 (Very much)*.

Table 4 presents social support, with the responses categorized according to type of support: emotional, concrete, informational, and spiritual. Parents and siblings were the most important sources of support. This was particularly true for women. Men relied significantly more often than women on sources outside the family for concrete support (18.4%, from Iraqis living in Sweden, for men vs. 10.9% for women) and informational support (7.7%, from members of associations and churches, for men, vs. 1.6% for women).

**Table 4:** Frequency and percentage for social support, by gender, in Sweden.

	**Male**		**Female**		**Total**		**Missing**
**Help from …**	***N***	**%**	***N***	**%**	***N***	**%**	
**Emotional support**							20
Partner/children	112	54.1	86	47	198	50.8	
Parents/siblings***	102	49.3	123	67.2	225	57.7	
Other relatives	29	14	26	14.2	55	14.1	
God	59	28.5	61	33.3	120	30.8	
No one	17	8.2	7	3.8	24	6.2	
**Concrete support**							19
Partner/children	83	40.1	69	37.5	152	38.9	
Parents/siblings ***	99	47.8	112	60.9	211	54	
Other relatives	53	25.6	49	26.6	102	26.1	
God	9	4.3	4	2.2	13	3.3	
People from Iraq in Sweden**	38	18.4	20	10.9	58	14.8	
No one**	36	17.4	19	10.3	55	14.1	
**Informational support**							19
Partner/children	92	44.4	83	45.1	175	44.8	
Parents/siblings**	109	52.7	115	62.5	224	57.3	
Other relatives	35	16.9	32	17.4	67	17.1	
God	21	10.1	12	6.5	33	8.4	
People from associations, churches***	16	7.7	3	1.6	19	4.9	
No one	34	16.4	30	16.3	64	16.4	
**Spiritual and religious support**							26
Partner/children	66	32.5	56	30.9	122	31.8	
Parents/siblings***	70	34.5	93	51.4	163	42.4	
Other relatives	11	5.4	13	7.2	24	6.3	
God	48	23.6	36	19.9	84	21.9	
No one	20	9.9	13	7.2	33	8.6	

*Chi–square tests for gender differences*,

*** *p < 0.01*,

** *p < 0.05*.

*Reporting those answers with responses of higher than 20% (0.2)*.

As to reported lack of support, 16.4% of the participants reported no support at all and an additional 14.1% no concrete support (Table 4). In the latter subgroup the gender difference was significant (17.4% for men, 10.3% for women). The level of perceived support from authorities (not in table) was even lower, ranging from 1.5% for emotional support to 6.1% for informational support.

In open responses, the respondents described the educational opportunities in Sweden as very positive, whereas work life was often regarded as discriminating and difficult. They generally appreciated the stability, equality, and justice they encountered in their new country. Concerning safety, one person said: “I feel much safer here than in Iraq. I have not been humiliated based on my Christian religion.” Another person was more ambivalent: “I feel safe, but sometimes I experience things that make me feel depressed, when I compare myself to a Sweden.” Some women reported facing discrimination for wearing a veil. Despite their perception of the situation in Sweden as better than in Iraq, and their attesting to feeling safe, some respondents were markedly concerned about physical security and criminality in society, which negatively affected their total sense of security.

Table 5 covers religiosity among participants. The results show significant gender differences, women responding that they were “very” religious more often than men (74% vs. 60.1%). As to religious activities such as fasting, a majority answered “sometimes” (26.1%) or “often” (33.3%), again with a significant difference between women and men. Another item, “how often do you pray?,” similarly showed a significant overrepresentation for women. A large majority, 341 persons (85.5%), of whom 181 were men and 160 women, responded that they belonged to a religion or religious denomination: Muslim (39.7%), 59 men and 76 women; Christian (33.2%), 62 men and 51 women; and Mandaean (26.8%), 57 men and 34 women. Asked about their image of God, a majority believed in a personal God (94.9%)−197 (52.8%) men and 176 (47.2%) women—whereas only 1,8% indicated adherence to what might be called an atheist orientation, explicitly excluding any belief in a personal God.

**Table 5:** Frequency and percentage for religiosity, meaning, safety, and gender equality, by gender (*N* = 410).

	**Men**		**Women**		**Total**		**Missing**
	***N***	**%**	***N***	**%**	***N***	**%**	
Belonging to a religion or religious denomination	181	53.1	160	46.9	341	85.5	11
How religious are you? Somewhat/a lot ***	122	60.1	134	74	256	66.7	26
Religious denomination**: Christian	62	54.9	51	45.1	113	33.2	70
Muslim	59	43.7	76	56.3	135	39.7	
Mandean	57	62.6	34	37.4	91	26.8	
I believe in personal god (opposite to belief in a spirit, or no belief at all)	197	52.8	176	47.2	373	94.5	17
Apart from weddings and funerals, about how often do you attend religious services/prayer services these days? Sometimes/often	131	62.4	133	71.5	264	66.7	14
How often do you fast? Sometimes/often ***	102	48.6	135	71.4	237	59.4	11
How often do you pray privately? Sometimes/often ***	119	56.7	143	75.7	262	65.7	11
How often, if at all, do you think about the meaning and purpose of life? Sometimes/often **	168	80	160	87.9	328	83.7	18
Do you feel safe and secure at home? Pretty/very much	197	90.8	175	92.1	372	91.4	3
Do you feel safe and secure in your neighborhood? Pretty/very much	182	85	166	87.8	348	86.4	7
On the whole, men make better political leaders than women do. Disagree/strongly disagree	137	63.4	133	69.9	270	66.3	3
A university education is more important for a boy than for a girl. Disagree/strongly disagree	189	87.9	178	92.7	367	90.2	3

*Chi-square tests for gender differences*,

*** *p < 0.01*,

** *p < 0.05*.

Table 6 presents traumatic experiences among the participants. There were significant differences between men and women, 63 (31.7%) vs. 41 (23.8%), respectively. While men more often responded that they had experienced traumatic situations, women reported having had more problems caused by the effects of traumas. The open-ended questions revealed that almost all trauma experiences (direct or indirect) were war-related, for example, explosions, public executions, kidnappings, threats, bombings, prison, assault, and torture. Other traumatic experiences related to traffic accidents, in- or outside of a war setting.

**Table 6:** Frequency and percentage for general health, trauma and resilience, by gender.

	**Men**		**Women**		**Total**	
	***N***	**%**	***N***	**%**	***N***	**%**
**Health**						
Excellent	35	17.07	22	12.09	57	14.73
Very good	50	24.39	36	19.78	86	22.22
Good	76	37.07	70	38.46	146	37.73
Fair	37	18.05	47	25.82	84	21.71
Poor	7	3.41	7	3.85	14	3.62
**Trauma**						
Difficult situation*	63	31.7	41	23.8	104	28
PTSD	21	10.2	22	12	43	11.1
**Resilience**						
CD-RISC 5+	106	53.8	78	45.6	184	50
CD-RISC 6+^*^	54	28.1	35	20.5	89	24.5

*Chi-square tests for gender differences*,

* *p < 0.1*.

*CD-RISC has cut-off levels at both 5+ and 6+*.

### Resilience

Table 7 presents self-assessment in personal and social areas and goals in life, both in Iraq and in Sweden. The ability to maintain a helpful social network deteriorated significantly after migration. The means and standard deviations for “socialization” and “network” in Iraq were *M* = 0.74, *SD* = 0.44 and *M* = 0.75, *SD* = 0.43, respectively, and in Sweden, *M* = 0.59, *SD* = 0.49 and *M* = 0.57, *SD* = 0.49, respectively. An overall significant ability to adapt to new situations was found nonetheless (Table 7). This effect was driven by a marked change among women as compared to men (*p* = 0.025). The women evaluated themselves as working more in Sweden than in Iraq, they worked both at home and were employed or pursued an education.

**Table 7:** Frequency and percentage for self-assessment in personal and social areas and goals in life, by gender and by country (*N* = 410).

	**Male**			**Female**		
	**Iraq**	**Sweden**	**Iraq-Swe Difference**	**Iraq**	**Sweden**	**Iraq-Swe Difference**
I (could) make friends	91	91		85	84	
	71.7%	63.6%	−8.0%	65.4%	59.6%	−5.8%
I could socialize with others/It is easy to socialize with others	101	89		108	77	
	76.5%	62.2%	−14.3%**	77.1%	57.5%	−19.7%***
I was/am confident in handling challenges in life	80	99		75	88	
	64.5%	71.7%	7.2%	63.0%	74.6%	11.6%
I was/am a hardworking person	109	95		79	104	
	70.3%	78.5%	8.2%	62.7%	80.6%	17.9%*
I was/am goal–oriented	81	93		79	92	
	69.8%	75.0%	5.2%	65.3%	74.8%	9.5%
I was/am a happy person	82	90		79	75	
	59.0%	70.3%	11.3%	62.2%	65.8%	3.6%
I could/can adapt to new situations	72	100		61	92	
	62.6%	70.9%	8.3%	54.0%	73.6%	19.6%**
I was/am a successful person	105	72		82	73	
	77.2%	69.2%	−8.0%	72.6%	68.9%	−3.7%
I had/have a large and helpful network of people around me	117	64		94	64	
	80.7%	55.7%	−25.0%***	73.4%	57.7%	−15.8%**

*Chi-square tests for country differences*.

*** *p < 0.01*,

** *p < 0.05*,

* *p < 0.1*.

The respondents reported that the influence of family, relatives, friends, and neighbors both in Iraq and Sweden was “positive” or “very positive.” The largest negative differences in this regard between Iraq and Sweden concerned the change in the rates of “positive” and “very positive” influence of neighbors (reported in 84.3% cases in Iraq and 73.8% in Sweden). The smallest change was observed for the influence of family (from 97.4% in Iraq to 96.7% in Sweden).

Differences between men and women in experienced social support before and after migration were stark. For men there were no significant differences between living in Iraq or Sweden, neither in any of the items referring to family, friends, religion, and spirituality, nor in items about work and school in stable times (Table 3). There was, however, a significant decrease in the importance of family for females. Gender differences were also significant with regard to the impact of religion and spirituality (*p* = 0.026): women more often than men tended to rely on religion in making sense of life—in Iraq as well as in Sweden.

Many respondents described problems with health (42% of the total sample, of which almost two-thirds were men), finances (48%, of which more than half were men), and discrimination (31%, of which more than half were men) as barriers to achieving their goals in life. Men more often emphasized health issues than did women (*p* = 0.047).

Table 8 presents perceptions of illness. The personal explanations are categorized as social-situational, emotional-developmental, medical-biological, or religious-spiritual (*cf*. Estroff et al., 1991). Only 11.1% of the respondents identified explanations for mental health problems belonging to a single category.

**Table 8:** Frequency and percentage for types of illness explanations for mental health problems, by gender.

	**Men**		**Women**		**Total**	
	***N***	**%**	***N***	**%**	***N***	**%**
Medical/clinical	141	67.8	123	65.8	264	66.8
Emotional/developmental	177	85.1	165	88.2	342	86.6
Social/situational	184	88.5	165	88.2	349	88.4
Religious/spiritual **	33	15.9	45	24.2	78	19.7
Other cause	10	4.8	5	2.7	15	3.8

*Chi-square tests for gender differences*,

** *p < 0.05*.

*Missing: 15*.

Social and situational problems (in total 88.4%), foremost financial difficulties and unemployment (68.4%), were the most commonly given explanations of mental ill health (Table 8). Difficulties directly related to being a refugee were less common: 29.6% explained mental health problems by having been exposed to violence or threats, 24.1% by having had to flee from their country of origin, and 13.9% for persecution because of political or religious convictions.

Emotional and developmental explanations (86.6%) were almost as common as social explanations. Problems in close relationships, failures in life, and the death of or separation from a significant other were frequently mentioned. Death or separation was the most common explanations for women (52.4%), who significantly differed from the men in this respect (*p* = 0.002).

Medical or biological explanations of psychological problems or mental health issues were indicated by 66.8% of the respondents. Significantly more women than men believed that biochemical changes in the body caused mental health problems (*p* = 0.01). Religious or spiritual explanations were given by 19.8% of the respondents. Significantly more women than men explained mental health problems as caused by religious and spiritual forces.

Overall, particularly for women, religion was a significant supportive factor of coping with challenges of everyday life (see Table 3). Even though the support from the family had decreased after the migration to Sweden, the help from friends and religion increased. The most salient, though not statistically significant, positive change for women's social adaptation in the new country was, however, their utilization of support in school and in the workplace. Nature or being outdoors helped coping with difficult situations for both genders. There was a highly significant change in this respect, for both men and women, between Iraq and Sweden.

Table 6 presents results on self-perceived resilience as reflected on the CD-RISC scale. The median for the overall resilience score was 5.5, which is at the lower level of the scale. The number who met the criterion for resilience when using the cut-off 6+, was 54 (28.1%) for men, and 35 (20.5%) for women. A similar pattern was found using the cut-off 5+.

### Health and Acculturation Outcomes

Almost 75% of the respondents evaluated their health as either “good,” “very good,” or “excellent” (Table 6). As is common for self-rated health, women (M = 2.9, SD = 1.04, where “excellent” was coded as 1 and “poor” as 5) evaluate their health slightly worse than men, though these differences are not significant (M = 2.66, SD = 1.07, *p* = 0.31). Additionally, 36.3% (71 men and 66 women) reported having experienced moderate to extreme pain that interfered with their work during the past 4 weeks. Problems with work or other regular daily activities caused by emotional problems (such as feeling depressed or anxious) were reported by 31.8% (61 men and 59 women) or to not carrying out work or other activities as diligently as usual by 27.5% (47 men and 52 women). Forty-three participants (11.1%, 21 men and 22 women) met the criteria for PTSD.

The ethnic labels used by the respondents were very diverse, frequently employing combinations of national and ethnic or religious subcategories, such as Iraqi- Arab-, or Suriani- (indicating Assyrian-Christian), Chaldean, and Kurdish. The majority of the respondents (both men and women, 68 and 63%, respectively) felt that they belonged both to their culture of origin as well as to Swedish culture and society; about 50% clarified that this double sense of self was an integrated (relatively conflict-free) experience. Yet at least half of the respondents (50.5%) reported having been treated unfairly based on their ethnic background or lack of language skills.

Acculturation outcomes were observed by asking about gender values, education, and family roles. A third of the sample responded that men make better political leaders than women (Table 5). As to the statement that a university education is more important for a boy than for a girl, about 10% agreed (checking either “strongly agree” or “agree,” with women showing stronger disagreement than men).

Finally, both men and women perceived that their role in the family had changed “somewhat” in Sweden compared to how it was in Iraq.

## Discussion

Our study results with this group of Iraqi individuals, who had lived longer in their new country than subjects in comparable studies, illuminated the role played by social and psychological determinants of mental health, both pre- and post-migration, as well as the association of self-evaluated health, resilience, and benign acculturation processes among them. The widespread, dynamic bicultural, to varying degrees, sense of identity in our sample as in other immigrants should be acknowledged in integration policies by providing space for the realization and interaction of both the original and the new host culture. Similarly our finding that women showed greater adaptability and acculturation, yet at the same time lower mental health, which highlights the stress of the refugee's acculturation, deserves being taken to heart in integration policies. The family remained the most important source of social support in the new country, while support from other sources deteriorated.

### A Lack of Social Support

A substantial proportion of the respondents did not receive either informational or emotional support from anyone. Combined with the fact that support from the authorities was generally perceived as very low, this finding indicates an unmet basic need and a challenge for Swedish society.

In earlier studies, other scales than those that we applied were used to rate social support, which makes comparisons difficult. We note, however, that in two studies social support was found to be low for both asylum seekers and refugees in general, with lower figures for asylum seekers (Gerritsen et al., 2006; Tinghög et al., 2016). The relatively high levels of perceived social support by the respondents in our study may, we contend, be explained by their relatively long time of residence in Sweden.

### Aspects of Resilience

Measuring the level of resilience with the instrument CD-RISC, we found that the median resilience level among the Iraqi refugees was low, 5.5. Women scored lower than men, regardless of cut-off level, notwithstanding that they showed higher levels of self-efficacy and adaptation. There was an apparent discrepancy between the relatively low level of resilience measured by the two CD-RISC items and the high level of resilience grounded on measures of coping, meaning, and goals in life. This difference may reflect that the CD-RISC items reflect individual, intrapsychic qualities of resilience and low scores are coupled to mental ill-health concerns, whereas the coping-, meaning-, and life-goal items are grounded on interpersonal and social dimensions. Recognizing the interconnectedness of meaning and resilience (Panter-Brick and Eggerman, 2012), we like Ungar (2008) and Kirmayer et al. (2011), combined the two perspectives.

The low level of resilience measured with CD-RISC may nonetheless depend not only on psychological and individual network resources, but also on societal factors. Our results show well-known post-migration problems on that level, for example, unemployment, financial problems, reduced social networks, and discrimination. These findings point to the importance of addressing the socio-economic realities of refugees in order to strengthen their collective and individual resilience.

Cultural aspects of resilience were addressed by questions on perception of illness. That almost 90 % of the participants presented more than one “cause” of mental illness agrees with the findings of other research that show that personal explanations are not as fixed as might expected, but vary with context and cultural norms on how to communicate personal suffering (Lewis-Fernández et al., 2014). The majority of the respondents in or study gave social and situational explanations of mental illness. The finding that few respondents presented refugee-related explanations, such as traumatic experiences, concurs with the observation by Shannon et al. (2012) that a reason that their research subjects, refugee patients in primary care in the Midwest USA, avoided talking to general practitioners about trauma was that they didn't link trauma and ill-health. In all, our findings may also reflect the greater impact of general socioeconomic stressors. The results point to the need for clinicians to beware of stereotyping their patients' cultural perceptions and instead be prepared to explore them together with the patient.

### Self-Perceived Health

The level of perceived health in this study is higher than in a comparable study in Sweden (Sundell Lecerof, 2010) as well as in a study on the Mandaean population in Australia (Nickerson et al., 2009). Our results also showed relatively low levels of PTSD (cf. Fazel et al., 2005). Most likely, PTSD in a population of migrants reflects its stratification in terms of subgroups such as war veterans and refugees of different ages, cultural backgrounds, and diagnoses.

Cultural differences within our sample and the fact that it was a convenience sample may constitute a threat to the validity of our findings. There may, for example, have been an unrecognized underlying reluctance among the participants to admit psychological symptoms or talk openly about traumatic experiences. These circumstances invite questions about host-country-specific factors, which would need to be investigated further.

### Gender Differences in Acculturation

The women in our sample were significantly better adapted and evaluated themselves as more hard-working in Sweden than in Iraq. The burden of work responsibilities at home as well as in a workplace was experienced as positive by some but not all of the women. Different circumstances and concerns appeared to influence how the shift of family roles was perceived. The high degree of adaptability and self-efficacy of many women in our study may be understood against the backdrop of a pre-migration renegotiation of gender roles and the economic expansion in Iraq during the 1970s, during which period women were economically productive (Nelson et al., 2016). These observations on gender-specific patterns of adaptation are important for integration policies and programs, which need to be sensitive to differences in the acculturation process between women and men.

Similar to Miller and Hoffmann (1995) we found a significantly stronger religious commitment and activity among the studied women compared to the men. Other studies of immigrants have found different patterns: while religious affiliation is often higher among females, religious activity in terms of weekly attendance at religious services tends to be equally stable among men and women or show a higher rate among men (Finke and Stark, 1992; Van Tubergen, 2006). Men's attendance at religious activities or meetings is often attributed to the specific religious group they belong to, for example, Islam (Van Tubergen, 2006). In our study the local religious community appeared to be a welcoming context that positively affected the religious activity of newcomers, mainly Christian worshippers, both men and women.

### Theoretical Reflection

Use of the two-domain mental health model orientation was important for tracking dimensions of both mental ill-health as well as well-being in the study population. This orientation allowed for a more complex and multi-dimensional understanding of resilience to be explored, as well as gender differences in exploring different social determinants of health and mental health. Mental ill-health concerns and well-being resources often co-exist and can dynamically influence each other (Kirmayer et al., 2011; Kalra et al., 2012; DeMarinis, 2014; Forsman et al., 2015; DeMarinis and Boyd-MacMillan, 2019; Boyd-MacMillan and DeMarinis, 2020). Mapping both areas and their interaction is important for clinicians' diagnosing and treatment planning processes.

### Methodological Reflections: Strengths and Limitations

For this study we were able to recruit a fairly large and diverse number of participants, despite initial difficulties in reaching out to refugees or newly arrived immigrants. Working through community networks or key-persons increased the level of trust, and facilitated the distribution of the survey. We found that trust usually could be established by respecting the participants' opinions, maintaining a neutral political and religious position, and demonstrating knowledge and competence in the participants' culture. We were also able to reach non-Muslim minority groups from Iraq as well as women and younger age groups, groups that are sometimes underrepresented in research. Collaborating with an Arabic-speaking research assistant with cultural knowledge of the field facilitated recruitment—notwithstanding the suspiciousness and lack of trust that sometimes came with the current political, social, and sectarian conflicts and fault lines of the Arabic-speaking region (the Middle East and North Africa).

There are some limitations to our study. The use of convenience sampling limits the representativity of our sample and the generalizability of our results: the observed high rates of self-perceived health, the low levels of PTSD as well as of aspects of resilience, compared to what was reported in studies of other refugee samples, may reflect a selection bias. But there are also arguments in favor of convenience sampling as appropriate for exploratory research in which new theoretical ideas and hypotheses may be generated (Bryman, 2015). Our aim has not been to present the mental health of Iraqi refugees at large, but to give a rich description of a time-specific period in Sweden and a not so small, diverse population of Iraqi refugees (*N* = 410) from a wide range of aspects. We did not aspire to cover the larger population of Iraqi refugees, who migrated at different time periods during the late 20th and early 21st centuries, but focused on a delimited population of Iraqis, who arrived soon after the year 2000. We hope the results might be useful in other contexts, as new refugee populations from other conflicted regions continue to face similar issues.

Another limitation was technical. The survey involved many questions and some complicated instruments, which may have resulted in lower reliability. An example of a disruptive constellation of questions was when we asked the respondents to select only one alternative response to one type of question and this was followed by questions with several response alternatives. There were indications in the material that this approach led to misunderstandings.

## Conclusions

The group of Iraqi individuals in this study, who had lived longer in their new country than subjects of comparable studies, showed relatively low levels of general mental health problems, including PTSD. Nevertheless, they remained vulnerable in some respects, notably with regard to individual and long-term psychological resilience.

Unemployment was high among the participants, even after a long period of living in Sweden. Social networks outside the family were weak, and authorities were not perceived as supportive. Authorities therefore need to address the challenge of answering the unmet needs of informational and concrete support.

Women evinced poor mental health in spite of signs of strong adaptation in other respects. Clinicians need to be aware of the risks and variations of psychosocial maladaptation and ill-health in refugees in general and women in particular.

The post-migration situation and the refugee's ways of managing need to be better understood in order to avoid too schematic views of the acculturation process. Variations in illness perception point to the importance for clinicians to avoid stereotyping in the encounter with refugee patients. The exploration of the individual's social world and subjective perceptions is crucial. The cultural formulation interview in the DSM-5 is a supportive tool in that exploration (Lewis-Fernández et al., 2014).

The gender differences found in our study should be viewed within a context of acculturation, where several determining single factors and patterns of interacting social and individual strengths and vulnerabilities play a central role. In a highly secularized society such as that in Sweden, with a strong individual goal-orientation and focus on self-reliance and gender equality, Iraqi women's comparatively strong ability to adapt to new situations and to evaluate themselves as hard-working members of Swedish society, may reflect—or have provided a basis for—a positive acculturation process. The same societal context may have been a greater challenge for the men. This difference may impact family acculturation processes in subsequent generations too.

### Key Policy Messages

-It is important to provide women and men with practical resources and frameworks for self-fulfillment in education and work, at the same time as they are supported in using cultural coping mechanisms, such as religion and nature.
-In the framework of social integration in work and education, caregivers need to be aware of the high prevalence of negative psychosocial and mental ill-health among women and of the differing needs for support in women and men.
-Culturally and locally adapted social information and support by authorities, together with support to maintain links with and to form new social networks is important for integration of newcomers in society.

## Data Availability Statement

The datasets presented in this article are not readily available because by the time of the data collection, participants were not informed about the possibility of making data available to outside researchers. Requests to access the datasets should be directed to Önver Cetrez, cetrez@teol.uu.se.

## Ethics Statement

The studies involving human participants were reviewed and approved by Swedish Ethical Review Authority. The patients/participants provided their written informed consent to participate in this study.

## Author Contributions

ÖÇ, VD, MS, and MG contributed conception and design of the study. ÖÇ organized the database and wrote the first draft of the manuscript. LB performed the statistical analysis. VD, MS, DT, and LB wrote sections of the manuscript. ÖÇ, VD, MS, DT, and LB contributed to manuscript revision, read, and approved the submitted version. All authors contributed to the article and approved the submitted version.

## Conflict of Interest

The authors declare that the research was conducted in the absence of any commercial or financial relationships that could be construed as a potential conflict of interest.
