# Supplementary material for: A Public Mental Health Study Among Iraqi Refugees in Sweden: Social Determinants, Resilience, Gender, and Cultural Context
Source: Front Sociol. 2021 Apr 26;6:551105. doi: 10.3389/fsoc.2021.551105 (PMC8109031; doi:10.3389/fsoc.2021.551105)
Supplement: Supplementary file 1 [file Data_Sheet_1.PDF]

QUESTIONNAIRE FOR IRAQIS IN SWEDEN

All rights reserved. No part of this document may be reproduced or transmitted in any form, or by any means, electronic or mechanical, including photocopying, or by any information storage or retrieval system, without permission in writing from Dr. Cetrez at cetrez@teol.uu.se. *Copyright © 2013, by Önver A. Cetrez, Ph.D., Valerie DeMarinis, Ph.D., Manuel Fernandez, MD, Maria Sundvall, MD.*

BACKGROUND QUESTIONS

1. What is your sex?
   - 1❑ Male
   - 2❑ Female

2. What is your year of birth? 19____
3. In what city are you currently living? ________________

4. What year did you arrive to Sweden? ______

5. What is your current relationship situation?
   - 1❑ Married
   - 2❑ Living together as married
   - 3❑ Divorced
   - 4❑ Engaged
   - 5❑ Widowed
   - 6❑ Single

6. Do you have children (no matter what age)?
   - 1❑ No
   - 2❑ If yes, how many are living at home? ……………..

7. What is the highest educational level that you have attained?
   - 1❑ No formal school education at all
   - 2❑ Primary school—number of years__
   - 3❑ Secondary school (”gymnasium”)—number of years __
   - 4❑ University – number of years__

8. In which country is your highest educational level attained? …………………………….

9. Are you working? If yes, and working more than one job, answer only for the main job:
   - 1❒ Employed
   - 2❒ Self employed
   - 3❒ Retired/pensioned
   - 4❒ Housewife
   - 5❒ Student
   - 6❒ Unemployed
   - 7❒ Unemployed, I receive economic support from: ………………………………
   - 8❒ Other (please explain)………………………………………

10. Have you during the last 12 months had difficulties in managing the standard costs for food, rent, bills, etc.?
    - 1 ❒ No
    - 2 ❒ Yes, on one occasion
    - 3 ❒ Yes, on several occasions

11. Which languages do you speak in the following situations (you can provide multiple alternatives)

A. At home …………………………………………………………………………..
B. With friends………………………………………………………………….
C. At work……………………………………………………………………….
D. In your free time………………………………………………………….
E. On internet………………………………………………………………….

12. How would you rate your Swedish language competency in general?

Very strong	Fairly strong	Fairly weak	Very weak
1❑	2❑	3❑	4❑

13. Do you belong to a religion or religious denomination? If yes, which one?

0 ❐ No, do not belong to a denomination
1 ❐ Yes, please give name of the tradition……………………

14. When you present yourself, which classification do you give for your ethnicity/ethnic group?
………………………………………………………………………………

15. How much do you identify yourself with the ethnicity /ethnic group you indicated above?

Not at all	A little	Somewhat	Very Much
1❐	2❐	3❐	4❐

16. How much do you feel a part of the Swedish society?

Not at all	A little	Somewhat	Very Much
1❐	2❐	3❐	4❐

17. How religious are you?

Not at all	A little	Somewhat	Very Much
1❐	2❐	3❐	4❐

18. Which of these fits with what you believe? (choose only one alternative)
1❑ I believe in a personal God
2❑ I believe in a spirit or life force
3❑ I don't believe in a spirit, life force, nor a personal God
4❑ I don't know what to believe
5❑ Other (please describe)…………………………………………………..

	Never	Rarely	Some-times	Often
19. How often, if at all, do you think about the meaning and purpose of life?	1❑	2❑	3❑	4❑
20. How often do you fast?	1❑	2❑	3❑	4❑
21. How often do you pray privately?	1❑	2❑	3❑	4❑
22. Apart from weddings and funerals, about how often do you attend religious services/prayer services these days?	1❑	2❑	3❑	4❑

	Never/ Almost never	Seldom	Often	Very often
23. How often do people treat you unfairly or negatively because of your ethnic or religious background?	1❑	2❑	3❑	4❑
24. How often do people treat you unfairly or negatively because of your Swedish language skills?	1❑	2❑	3❑	4❑

	Not at all	Little	Some-what	Much
25. Do you feel safe and secure at home?	1❑	2❑	3❑	4❑
26. Do you feel safe and secure in your neighbourhood?	1❑	2❑	3❑	4❑

27. For the following statements, can you specify how strongly you agree or disagree with each.

	Strongly agree	Agree	Disagree	Strongly disagree
A. On the whole, men make better political leaders than women do.	1❑	2❑	3❑	4❑
B. A university education is more important for a boy than for a girl.	1❑	2❑	3❑	4❑

28. Please, take a moment to comment, with your own words, the experiences related to the above questions on contact with the Swedish society, changes in life, discrimination, and safety.

…………………………………………………………………………………………

…………………………………………………………………………………………

…………………………………………………………………………………………

29. Think back to your most stable time in Iraq. Indicate two of the alternatives that described you best as a person, and two that described you least:	Described me best	Described me least
A. I could make friends	❑	❑
B. I could socialize with others	❑	❑
C. I was confident in handling challenges in life	❑	❑
D. I was a hardworking person	❑	❑
E. I was goal-oriented	❑	❑
F. I was a happy person	❑	❑
G. I could adapt to new situations	❑	❑
H. I was a successful person	❑	❑
I. I had a large and helpful network of people around me	❑	❑

30. What describes you best and least as a person, now in Sweden?	Described me best	Described me least
A. I have easy making friends	❑	❑
B. I have easy socializing with others	❑	❑
C. I have confidence in handling challenges in life	❑	❑
D. I am a hardworking person	❑	❑
E. I am goal-oriented person	❑	❑
F. I am a happy person	❑	❑
G. I have easy to adapt to new situations	❑	❑
H.I am a successful person	❑	❑
I. I have a large and helpful network of people around me	❑	❑

31. What influence did the following people have on you/how you thought about yourself, during your most stable time in Iraq? If you didn't have any, choose the last option.

	Very bad	Somewhat bad	Somewhat good	Very good	Didn't have any
A. Family	1❑	2❑	3❑	4❑	❑
B. Relatives	1❑	2❑	3❑	4❑	❑
C. Friends	1❑	2❑	3❑	4❑	❑
D. Neighbours	1❑	2❑	3❑	4❑	❑

32. What influence do the following persons have on you/how you think about yourself, now in Sweden? If you don't have any, choose the last option.

	Very bad	Somewhat bad	Somewhat good	Very good	Don't have any
A. Family	1❑	2❑	3❑	4❑	❑
B. Relatives	1❑	2❑	3❑	4❑	❑
C. Friends	1❑	2❑	3❑	4❑	❑
D. Neighbours	1❑	2❑	3❑	4❑	❑

33. Please rate how much the following helped you to make sense/gave your life meaning during your most stable period in Iraq?

	Family	Friends	Religion/ Spirituality	Work/ School	Other (specify) …………
Helped very much	10❑	10❑	10❑	10❑	10❑
	9❑	9❑	9❑	9❑	9❑
	8❑	8❑	8❑	8❑	8❑
	7❑	7❑	7❑	7❑	7❑
	6❑	6❑	6❑	6❑	6❑
	5❑	5❑	5❑	5❑	5❑
	4❑	4❑	4❑	4❑	4❑
	3❑	3❑	3❑	3❑	3❑
	2❑	2❑	2❑	2❑	2❑
Did not help at all	1❑	1❑	1❑	1❑	1❑
Not relevant	0❑	0❑	0❑	0❑	0❑

34. Please, rate how much the following help you to make sense/gives your life meaning here in Sweden:

	Family	Friends	Religion/ Spirituality	Work/ School	Other (specify) …………
Helps very much	10❑	10❑	10❑	10❑	10❑
	9❑	9❑	9❑	9❑	9❑
	8❑	8❑	8❑	8❑	8❑
	7❑	7❑	7❑	7❑	7❑
	6❑	6❑	6❑	6❑	6❑
	5❑	5❑	5❑	5❑	5❑
	4❑	4❑	4❑	4❑	4❑
	3❑	3❑	3❑	3❑	3❑
	2❑	2❑	2❑	2❑	2❑
Doesn't help at all	1❑	1❑	1❑	1❑	1❑
Not relevant	0❑	0❑	0❑	0❑	0❑

35. To what extent during your most stable **time in Iraq** did the following help you in coping with any difficult situation you have faced?

	Family	Friends	Religion/ Spirituality	Work/ School	Being out in Nature	Other (specify) …………
Very much	10❑	10❑	10❑	10❑	10❑	10❑
	9❑	9❑	9❑	9❑	9❑	9❑
	8❑	8❑	8❑	8❑	8❑	8❑
	7❑	7❑	7❑	7❑	7❑	7❑
	6❑	6❑	6❑	6❑	6❑	6❑
	5❑	5❑	5❑	5❑	5❑	5❑
	4❑	4❑	4❑	4❑	4❑	4❑
	3❑	3❑	3❑	3❑	3❑	3❑
	2❑	2❑	2❑	2❑	2❑	2❑
Not at all	1❑	1❑	1❑	1❑	1❑	1❑
Not relevant	0❑	0❑	0❑	0❑	0❑	0❑

36. To what extent here in Sweden do the following help you in coping with any difficult situation you are facing?

	Family	Friends	Religion/ Spirituality	Work/ School	Being out in Nature	Other (……….)
Very much	10❑	10❑	10❑	10❑	10❑	10❑
	9❑	9❑	9❑	9❑	9❑	9❑
	8❑	8❑	8❑	8❑	8❑	8❑
	7❑	7❑	7❑	7❑	7❑	7❑
	6❑	6❑	6❑	6❑	6❑	6❑
	5❑	5❑	5❑	5❑	5❑	5❑
	4❑	4❑	4❑	4❑	4❑	4❑
	3❑	3❑	3❑	3❑	3❑	3❑
	2❑	2❑	2❑	2❑	2❑	2❑
Not at all	1❑	1❑	1❑	1❑	1❑	1❑
Not relevant	0❑	0❑	0❑	0❑	0❑	0❑

37. What worried you most, during your most difficult time in Iraq?

	Family	Friends	Work/ School	Safety	Other (specify) …………
Very much	10❑	10❑	10❑	10❑	10❑
	9❑	9❑	9❑	9❑	9❑
	8❑	8❑	8❑	8❑	8❑
	7❑	7❑	7❑	7❑	7❑
	6❑	6❑	6❑	6❑	6❑
	5❑	5❑	5❑	5❑	5❑
	4❑	4❑	4❑	4❑	4❑
	3❑	3❑	3❑	3❑	3❑
	2❑	2❑	2❑	2❑	2❑
Not at all	1❑	1❑	1❑	1❑	1❑
Not relevant	0❑	0❑	0❑	0❑	0❑

38. What worries you most, now in Sweden?

	Family	Friends	Work/ School	Safety	Other (specify) …………
Very much	10❑	10❑	10❑	10❑	10❑
	9❑	9❑	9❑	9❑	9❑
	8❑	8❑	8❑	8❑	8❑
	7❑	7❑	7❑	7❑	7❑
	6❑	6❑	6❑	6❑	6❑
	5❑	5❑	5❑	5❑	5❑
	4❑	4❑	4❑	4❑	4❑
	3❑	3❑	3❑	3❑	3❑
	2❑	2❑	2❑	2❑	2❑
Not at all	1❑	1❑	1❑	1❑	1❑
Not relevant	0❑	0❑	0❑	0❑	0❑

39. What do you think are the underlying challenges for being able to achieve ambitions? Check all that apply:

1❑ My own health
2❑Family problems
3❑ My educational background
4❑Specific group identity
5❑ Discrimination in society
6❑Economic problems
7❑ Other concerns: (specify)…………………………………………

	Not at all					Very much				
	1	2	3	4	5	6	7	8	9	10
40. How much has your role in your family here in Sweden changed from how it was in Iraq?	❑	❑	❑	❑	❑	❑	❑	❑	❑	❑

41. We would now like you to answer some questions concerning people who give you different kinds of support in daily life. For every question you can mark one box for the category of persons who support you on that issue. You may mark several boxes for every question. If you choose the answer "Other", we also ask you to specify who the relationship that person has to you. Choose among the following options:

❑ A. My partner/children	❑ B. My parents/My siblings	❑ C. Other relatives
❑ D. God/ a spiritual force	❑ E. People I know from Iraq who live in other countries	❑ F. Neighbours in Sweden
❑ G. Persons I have come to know through associations, churches etc.	❑ H. Authorities in Sweden	❑ I. Persons I have come to know in my daily activities (school, practice, work)
❑ J. People I know from Iraq (neighbours, friends, colleagues etc) who live in Sweden	❑ K. No one	❑ L. Other

1. Who comforts you when you are upset?
A❑ B❑ C❑ D❑ E❑ F❑ G❑ H❑ I❑ J❑ K❑
L❑ Other, if so describe who…………………………………………………………

2. Whom can you confide in?
A❑ B❑ C❑ D❑ E❑ F❑ G❑ H❑ I❑ J❑ K❑
L❑ Other, if so describe who…………………………………………………………

3. Who helps you with your mail when you are away?
A❑ B❑ C❑ D❑ E❑ F❑ G❑ H❑ I❑ J❑ K❑
L❑ Other, if so describe who…………………………………………………………

4. Who can lend you 1000 Swedish crowns, if you need it?
A❑ B❑ C❑ D❑ E❑ F❑ G❑ H❑ I❑ J❑ K❑
L❑ Other, if so describe who…………………………………………………………

5. Who can help you to fill in forms for authorities?
A❑ B❑ C❑ D❑ E❑ F❑ G❑ H❑ I❑ J❑ K❑
L❑ Other, if so describe who…………………………………………………………

6. Whom can you consult if you have to make an important decision?
A❑ B❑ C❑ D❑ E❑ F❑ G❑ H❑ I❑ J❑ K❑
L❑ Other, if so describe who…………………………………………………………

7. Who can support you if you want to perform activities to give you spiritual strength?
A❑ B❑ C❑ D❑ E❑ F❑ G❑ H❑ I❑ J❑ K❑
L❑ Other, if so describe who…………………………………………………………

8. Who is the person supporting you most at present? (Indicate only one alternative.)
A❑ B❑ C❑ D❑ E❑ F❑ G❑ H❑ I❑ J❑ K❑
L❑ Other, if so describe who…………………………………………………………

42. Which of the following factors do you think could cause someone mental health problems?
Mark the boxes for the five causes that you consider the most important. If you mark "Other cause" we ask you to answer the question "which" on the same line.

❑1. Chemical changes in the body
❑2. Conditions when you grow up
❑3. Financial difficulties, unemployment
❑4. The evil eye/ other harm through spiritual forces
❑5. Heredity
❑6. Problems in close relations; conflicts at home
❑7. The death of a significant person, separation from a significant person
❑8. To have been exposed to violence or threat of violence
❑9. Brain damage
❑10. Side effects of medication
❑11. Failures in life
❑12. Housing problems
❑13. To be forced to flee from your country
❑14. God's punishment, a spiritual trial/test
❑15. Alcohol and drugs
❑16. Jinn
❑17. Conflicts at work/in school
❑18. Serious physical sickness, Accident with bodily harm
❑19. A normal crisis in the development of young persons
❑20. Persecution because of political or religious convictions
❑21. Other cause – in that case please describe?---

43. Where would you recommend a person you know in Sweden to seek help if he or she has mental health problems? Mark the box for what you would recommend primarily, secondarily and thirdly.

		1st hand	2nd hand	3rd hand
A.	Emergency ward in the closest hospital	❑	❑	❑
B.	Private physician	❑	❑	❑
C.	Private psychiatrist or psychologist	❑	❑	❑
D.	The general practitioner at the health centre	❑	❑	❑
E.	The social worker/psychologist at the health centre	❑	❑	❑
F.	Spiritual guide (priest, imam etc)	❑	❑	❑
G.	Traditional healer using herbal medication	❑	❑	❑
H.	The outpatient unit of the mental health services at the closest hospital	❑	❑	❑
I.	A doctor who talks your own language	❑	❑	❑
J.	The unit of occupational medicine at the workplace or school health	❑	❑	❑
K.	The pharmacy	❑	❑	❑
L.	Another (please describe)…………………………	❑	❑	❑

44. Have you visited any of the following health care providers during last year? Indicate in that case how you perceived the contact. If you have had no contact, choose "Not visited"

	Very Negative	Fairly negative	Fairly positive	Very positive	Not visited
A. Health centre – general practitioner	1❑	2❑	3❑	4❑	❑
B. Health centre – district nurse	1❑	2❑	3❑	4❑	❑
C. Health centre – social worker/ psychologist	1❑	2❑	3❑	4❑	❑
D. The unit of parental care	1❑	2❑	3❑	4❑	❑
E. Emergency ward in hospital	1❑	2❑	3❑	4❑	❑
F. Outpatient unit in hospital (not in the mental health services)	1❑	2❑	3❑	4❑	❑
G. Outpatient unit in the mental health services	1❑	2❑	3❑	4❑	❑
H. Private physician	1❑	2❑	3❑	4❑	❑
I. Physiotherapist (private, in health centre, in hospital)	1❑	2❑	3❑	4❑	❑
J. Psychotherapist	1❑	2❑	3❑	4❑	❑
K. Unit of occupational medicine or school health	1❑	2❑	3❑	4❑	❑
K. Not visited health care but talked to doctor or nurse on telephone	1❑	2❑	3❑	4❑	❑

Connor-Davidson Resilience Scale 2 (CD-RISC 2)

*For each item, please mark an "**x**" in the box below that best indicates how much you agree with the following statements as they apply to you over the last **month**. If a particular situation has not occurred recently, answer according to how you think you would have felt.*

		not true at all (0)	rarely true (1)	sometimes true (2)	often true (3)	true nearly all the time (4)
45 A	I am able to adapt when changes occur.	❑	❑	❑	❑	❑
45 B	I tend to bounce back after illness, injury, or other hardships.	❑	❑	❑	❑	❑

46. In general, would you say your health is:

Excellent ❑1 Very good ❑2 Good ❑3 Fair ❑4 Poor ❑5

47. The following two questions are about activities you might do during a typical day. Does YOUR HEALTH NOW LIMIT YOU in these activities? If so, how much?

A. MODERATE ACTIVITIES, such as moving a table, pushing a vacuum cleaner, bowling, or playing golf:
Yes, Limited A Lot ❑1 Yes, Limited A Little ❑2 No, Not Limited At All ❑3

B. Climbing SEVERAL flights of stairs:
Yes, Limited A Lot ❑1 Yes, Limited A Little ❑2 No, Not Limited At All ❑3

48. During the past 4 weeks, have you had any of the following problems with your work or other regular daily activities as a result of your physical health?

A. Accomplished less than you would like ❑1. Yes ❑2 No
B. Had difficulty performing the work or other activities ❑1. Yes ❑2 No

49. During the past 4 weeks, have you had any of the following problems with your work or other regular daily activities as a result of any emotional problems (such as feeling depressed or anxious)?

A. Accomplished less than you would like	❑1. Yes	❑2 No
B. Didn't do work or other activities as carefully as usual	❑1. Yes	❑2 No

50. During the past 4 weeks, how much did pain interfere with your normal work (including both work outside the home and housework)?

Not at all ❑1 A little bit ❑2 Moderately ❑3 Quite a bit ❑4 Extremely ❑5

51. How much of the time during the past 4 weeks . . .
A. Have you felt calm and peaceful?

❑1 All of the Time
❑2 Most of the Time
❑3 A Good Bit of the Time
❑4 Some of the Time
❑5 A Little of the Time
❑6 None of the Time

B. Did you have a lot of energy?

❑1 All of the Time
❑2 Most of the Time
❑3 A Good Bit of the Time
❑4 Some of the Time
❑5 A Little of the Time
❑6 None of the Time

C. Have you felt downhearted and blue?

❑1 All of the Time
❑2 Most of the Time
❑3 A Good Bit of the Time
❑4 Some of the Time
❑5 A Little of the Time
❑6 None of the Time

52. During the PAST 4 WEEKS, how much of the time has your PHYSICAL HEALTH OR EMOTIONAL PROBLEMS interfered with your social activities (like visiting with friends, relatives, etc.)?

❑1 All of the Time
❑2 Most of the Time
❑3 Some of the time
❑4 A Little of the Time
❑5 None of the Time

Primary Care PTSD Screen (PC-PTSD)

53.Have you experienced a difficult situation, e.g., serious accident, nature catastrophe, rape, war, abuse, torture?

❑No
❑Yes. If yes, describe the situation…………………………..

54. In your life, have you ever had any experience that was so frightening, horrible, or upsetting that, in the past month, you:

Have had nightmares about it or thought about it when you did not want to?
❑1. Yes ❑2 No

Tried hard not to think about it or went out of your way to avoid situations that reminded you of it?
❑1. Yes ❑2 No

Were constantly on guard, watchful, or easily startled?
❑1. Yes ❑2 No

Felt numb or detached from others, activities, or your surroundings?
❑1. Yes ❑2 No

Please check that you haven't forgotten to answer any questions!
THANK YOU FOR PARTICIPATION IN THIS STUDY!

Did anyone help you to fill in the questionnaire?

1❑No 2❑Yes (please write who helped you, a friend relative or other?)
…………………………………………………………………
